# Case of Punctured Wound of the Abdomen, Involving the Intestines

**Published:** 1852-06

**Authors:** 


*Case of Punctured Wound of the Abdomen, involving the Intestines—Artificial Anus—Recovery.*—About four years ago a young man, aged 25 years, in an affray was stabbed with a pocket-knife in the abdomen, on the left side, midway between the umbilicus and the anterior and superior spinous process of the ilium. The wound in the abdominal wall, as well as that of the intestine, was nearly an inch in breadth. As soon as he was stabbed, the intestine protruded, but it was immediately returned by a bystander. On his admission, the fæcal matter was voided freely by the external wound. Finding such to be the case, a warm poultice was merely applied; antiphlogistic regimen was enjoined; he was afterwards put under the constitutional influence of mercury, and kept quiet. The fæces gradually resumed the natural route; the external wound contracted, and by the end of a month it had closed entirely, when the man left the hospital, declaring, that save for his weakness, he never had felt better in his life. In this case, the adhesive inflammation had glued the intestine to the abdominal wall, so that the opening in the intestine continued to correspond with that in the wall. In a case where this did not occur, the attachment of the wounded gut to the wall, by means of suture, would be the only course to pursue; but in the example just cited, nature had obviated the necessity of any such proceeding. Perhaps, too, the natural position of the wounded gut had served to keep the two wounds in that apposition indispensably necessary for recovery.—*Ibid.*

*New Orleans, February*, 1852.

*More than* 150 *Gravel taken from the Bowels of a Dirt-Eating Child.* By Thomas Pollard, M. D., Richmond, Virginia.—July 14—Was called to see A. F., a child in his fifth year. Found him suffering with frequent tenesmus and pain in his bowels. Was informed by his parents that they had given him several doses of purgative medicine, which had brought from him *twenty-four gravel.* From subsequent inquiry it seemed he had swallowed these gravel through a morbid appetite, as he had several times been detected in eating dirt and pieces of pine bark. On introducing the finger into the rectum, I found it impacted with these little stones, the last operations of the medicine having failed to remove any of them. I proceeded to bring them away with the finger, and after getting all within the reach of my finger, counted fifty-four thus extracted. I could still touch more, and ordered a free dose of castor oil with eight drops of tinct. opium to allay the irritation and tenesmus, which was considerable, and which had been increased by the introduction of the finger and removal of the gravel. The swelling about the anus was considerable, and some blood was made to flow in the necessary manipulation used. There was also decided fever.

15—Rest was procured by the tinct. opium. The oil operated well towards the morning, and brought away 70 more of the gravel, the discharge of which was attended with much pain and some exhaustion. Directed sulph. morph. gr. $\frac{1}{8}$, and mucilages for drink.

16—Slept well after the administration of the morphine, and this morning is very comfortable. Discharged 4 more gravel. Altogether he has passed upwards of 150, some of which measured more than 1 inch in circumference, and few of which were smaller than the end of the little finger. The tenesmus and irritation gradually subsided, and convalescence was rapid. Directed that he should take sulph. iron as a tonic and subdue the morbid appetite which had given rise to the swallowing of these dainty bits. Was afterwards informed by his parents that the iron subserved the purpose intended, and that he never afterwards ate dirt. pine bark or gravel. I have generally found the sulph. iron to cure "dirt-eaters:" and I have been informed that on the large plantations of the south *copperas* is a very popular and successful remedy among the negro children who eat dirt.—*Stethoscope.*

